# Qualitative and Quantitative Evaluation of Blob-Based Time-of-Flight PET Image Reconstruction in Hybrid Brain PET/MR Imaging

**DOI:** 10.1007/s11307-015-0824-x

**Published:** 2015-01-30

**Authors:** Eva L. Leemans, Fotis Kotasidis, Michael Wissmeyer, Valentina Garibotto, Habib Zaidi

**Affiliations:** 1 Division of Nuclear Medicine and Molecular Imaging, Geneva University Hospital, 1211 Geneva 4, Switzerland; 2 Technical Medicine, University of Twente, 7522 NB Enschede, The Netherlands; 3 Geneva Neuroscience Center, Geneva University, 1205 Geneva, Switzerland; 4 Department of Nuclear Medicine and Molecular Imaging, University of Groningen, University Medical Center Groningen, 9700 RB Groningen, Netherlands

**Keywords:** PET/MRI, Image reconstruction, Blobs, Visual quality, Contrast, Noise

## Abstract

**Purpose:**

Many neurological diseases affect small structures in the brain and, as such, reliable visual evaluation and accurate quantification are required. Recent technological developments made the clinical use of hybrid positron emission tomography/magnetic resonance (PET/MR) systems possible, providing both functional and anatomical information in a single imaging session. Nevertheless, there is a trade-off between spatial resolution and image quality (contrast and noise), which is dictated mainly by the chosen acquisition and reconstruction protocols. Image reconstruction algorithms using spherical symmetric basis functions (blobs) for image representation have a number of additional parameters that impact both the qualitative and quantitative image characteristics. Hence, a detailed investigation of the blob-based reconstruction characteristics using different parameters is needed to achieve optimal reconstruction results.

**Procedures:**

This work evaluated the impact of a range of blob parameters on image quality and quantitative accuracy of brain PET images acquired on the Ingenuity Time-of-Flight (TOF) PET/MR system. Two different phantoms were used to simulate brain imaging applications. Image contrast and noise characteristics were assessed using an image quality phantom. Quantitative performance in a clinical setting was investigated using the Hoffman 3D brain phantom at various count levels. Furthermore, the visual quality of four clinical studies was scored blindly by two experienced physicians to qualitatively evaluate the influence of different reconstruction protocols, hereby providing indications on parameters producing the best image quality.

**Results:**

Quantitative evaluation using the image quality phantom showed that larger basis function radii result in lower contrast recovery (∼2 %) and lower variance levels (∼15 %). The brain phantom and clinical studies confirmed these observations since lower contrast was seen between anatomical structures. High and low count statistics gave comparable values. The qualitative evaluation of the clinical studies, based on the assessment performed by the physicians, showed a preference towards lower image variance levels with a slightly lower contrast, favoring higher radii and four iterations.

**Conclusion:**

This study systematically evaluated a number of basis function parameters and their quantitative and qualitative effect within PET image reconstruction in the context of brain imaging. A range of blob parameters can minimize error and provided optimal image quality, where the anatomical structures could be recognized but the exact delineation of these structures is simplified in scans with lower image variance levels and thus, higher radii should be preferred. With the optimization of blob parameters, the reconstructed images were found to be qualitatively improved (optimum parameters {d = 2.0375, alpha = 10.4101, radius = 3.9451}) as assessed by the physicians compared to the current clinical protocol. However, this qualitative improvement is task specific, depending on the desired image characteristics to be extracted. Finally, this work could be used as a guide for application-specific optimal parameter selection.

**Electronic supplementary material:**

The online version of this article (doi:10.1007/s11307-015-0824-x) contains supplementary material, which is available to authorized users.

## Introduction

A number of neurological diseases, such as epilepsy, dementia, or brain tumors, produce important alterations in small brain structures that are frequently not detectable using conventional anatomical imaging techniques [1]. Both magnetic resonance imaging (MRI) and x-ray computed tomography (CT) imaging provide detailed anatomic information with CT being faster, easily accessible, and able to visualize bone structures; however, MRI has several advantages over CT including the higher soft tissue contrast of MRI and the absence of radiation exposure in addition to providing useful functional information [2, 3]. Positron emission tomography (PET) imaging provides biochemical and molecular information at the cellular level. Therefore, hybrid or fusion PET/MR imaging could become the *de facto* standard procedure, offering unique capabilities for the clinical neuroimaging community and neuroscience research at large [4].

The recent development of MR-compatible PET components made hybrid PET/MR systems a reality. These systems facilitate co-registration of structural and functional images and enable simultaneous *in vivo* assessment of multimodality imaging probes. Such acquisition protocols potentially create a more convenient workflow for the patients as they undergo two examinations within a single scanning session. Moreover, multiple studies have shown potential clinical benefits of such hybrid imaging protocols, such as improved diagnostic accuracy [2–4]. However, the actual combination of PET and MRI faces two major technical challenges, namely reducing the potential interference between the two systems and developing reliable and robust MR-based attenuation correction (MRAC) schemes [5, 6].

Throughout this work, the Philips Ingenuity TOF PET/MR system, combining the Gemini TOF PET and the Achieva 3T X-series MRI scanners and arranged in a tandem geometry with a ∼3 m physical separation, was used, allowing sequential acquisition of PET and MR images. To minimize the interference between the two components, additional shielding of the photodetectors was used. MRI-based attenuation correction is performed through intensity-based tissue segmentation and classification into three categories: air, lung, and soft tissue [7]. Combination of these modalities in a single device could alter image characteristics, such as spatial resolution due to the magnetic field or bias due to the MR derived attenuation correction [8]. However, previous studies, using phantom and clinical data, have shown that the performance of the PET-subsystem was comparable to the Gemini TOF PET-CT system [9].

Quantitative and semi-quantitative metrics, such as the standardized uptake value (SUV), are often used for accurate clinical diagnosis, staging, restaging, disease monitoring, and assessment of response to treatment. In addition, it is important to differentiate small structures from neighboring structures due to their functional role in certain neurological diseases. This requires high spatial resolution with a high contrast between small brain structures and a high signal-to-noise ratio. These imaging characteristics impact visual interpretation as well as semi-quantitative and fully quantitative indices in brain PET imaging.

Traditionally, space modeling for image representation is carried out by means of cubic basis functions (voxels). Voxels are mainly used for simplicity reasons and due to the uniform sampling they provide for visualization. However, due to statistical noise, filtering is often required at the expense of reduced contrast and spatial resolution loss. The use of spherically symmetric basis functions (blobs) as opposed to voxels improves upon the former, with the blob-based reconstruction resulting in less image noise, without loss of resolution within a range of basis function parameters [10–13]. In addition, the smoother, overlaying spatial distribution of blobs will better represent the smoother biological transitions compared to the discontinuous sharp boundaries of voxels [14].

The blob density function can be altered by a set of parameters enabling the user to control the characteristics of the reconstructed image [11]. The chosen parameters could affect both the qualitative and quantitative performances of the reconstructed images [15]. Parameter selection depends on the task at hand, since the images should be individually optimized in terms of spatial resolution, contrast, and variance characteristics to suit a particular application. In addition, it is important to achieve comparable results in single- and multi-center trials by minimizing differences between image processing protocols [16]. Previous research investigated only a few blob parameter sets and assessed their effect on quantification [11, 12]. Hence, a detailed investigation of image reconstruction characteristics with a range of blob parameters is needed.

In this work, we quantitatively evaluated the impact of a range of blob parameters. Two different phantoms were used, simulating brain imaging applications. Using an image quality phantom, we systematically assessed the image contrast and noise characteristics. The Hoffman 3D brain phantom was also used to evaluate more realistically the different reconstruction parameters for brain imaging applications. The last part of this study was consisted of qualitative evaluation of clinical studies to assess which parameter set best represents the clinical need. The results of this study could be used to search and implement optimal reconstruction parameters for specific applications.

## Materials and Methods

### Theory

As mentioned above, the image space is modeled by a set of basis functions. Instead of cubic basis functions, other overlapping functions could be used and freely modified to reflect certain properties such as bias, variance, and spatial resolution. In this work, we use a common spherically symmetric basis function, the Kaiser–Bessel window function [14]. This function could be described as follows:

1 $$ b\left(m,\alpha, R;r\right) = \left\{\begin{array}{c}\hfill \frac{I_{\mathrm{m}}\left(\alpha \sqrt{1-{\left(\frac{R}{r}\right)}^2}\right)}{I_{\mathrm{m}}\left(\alpha \right)}{\left(\sqrt{1-{\left(\frac{R}{r}\right)}^2}\right)}^{\mathrm{m}},\ \mathrm{if}\ 0\le R\le r\hfill \\ {}\hfill 0,\kern13.75em \mathrm{otherwise}\hfill \end{array}\right. $$

where *m* is the order of the modified Bessel function (*I*_m_), *R* is the radial distance from the blob center, *r* is the blob radius, while the alpha (α) parameter affects the blob shape. The second order is selected (*m* = 2) as lower values give discontinuous blobs, while higher values create smoother basis functions but require longer computational *i*. The blob radius also influences the computational time, with a larger radius requiring more time since more lines of response (LORs) coincide with the blob. Alpha is the only parameter that does not influence the computational time. A large alpha results in an amplitude dropping close to the blob center and therefore a smoother transition.

Both alpha and radius parameters influence the spatial resolution defined as the full width at half maximum (FWHM) of the point spread function (PSF) and thus the variance characteristics of the reconstructed images. It should be kept in mind that the blob FWHM must be kept smaller than the FWHM of the scanner’s PSF. If this requirement is not met, the overshoot of high frequencies could cause ringing artefacts. To minimize the mean square error in the reconstructed image, the Fourier transform of the basis function must be zero (or as close to zero as possible) at a distance equal to multiples of the sampling distance of the image grid, as this ensures optimum overlapping between adjacent blobs. This requirement results in another parameter, the sampling distance (*d*). To fulfil this condition, Eq. 2 must be satisfied, which is a modified formula for a blob basis function grid based on the more generic formula for a cubic grid [11].

2 $$ \frac{r}{d/\sqrt{2}} = \frac{\sqrt{\alpha^2+{u}_{\mathrm{i}}^2}}{2\pi } $$

where *u*_i_ is the parameter at which the *i* ^*th*^ zero crossing of the 3-dimensional Bessel function occurs. The exact value of *u*_i_ can be obtained from lookup tables. When the conditions set in this equation are satisfied, the error of the images is minimized for a certain combination of parameters.

### Phantom Studies

Two different phantoms were used to evaluate the impact of the basis function parameters on image quality in brain PET imaging, namely an image quality phantom [17] and the more realistic Hoffmann 3D brain phantom [18]. The image quality phantom consists of a 20-cm diameter cylinder containing six spheres of different size (diameters ranging between 9.98 and 31.27 mm). The phantom was filled with 20 MBq of Fluorine-18 solution with a 5:1 ratio between the spheres and the warm background compartment.

The second phantom, the Hoffman brain phantom, provides an anatomical simulation of the radioisotope distribution found in the normal brain. Made out of sturdy plastic, it consists of 19 inserts made up of five thinner slices. Through thickness differences of the inner slices, a 4:1 ratio between the gray matter (GM) and white matter (WM) is mimicked. The phantom was filled with 43.9 MBq Fluorine-18 at the time of acquisition. To achieve a reliable MR signal with improved contrast, 350 mg copper sulfate was added to the fluorine solution.

The phantoms were carefully placed at the center of the field-of-view using built-in laser guides. All scans were acquired on the Philips Ingenuity TOF PET/MR system in list-mode format for 10 min (∼100 M events) and 45 min (∼430 M events) for the image quality phantom and the Hoffman 3D brain phantom, respectively (two separate scanning sessions). In order to assess the influence of counting statistics, the list mode data of the Hoffman phantom were rebinned to consider only the last 10 min of the scan, consisting of ∼85 M counts, thus mimicking counting statistics on the low count side of a typical brain scan.

### Clinical Studies

Four clinical brain studies of patients referred for dementia were used for visual qualitative evaluation. The patients were scanned using the usual protocol applied in clinical routine (waiting in a darkened room, low ambient noise level 15 min prior to injection, and scanning in supine position without the use of head fixation devices). The patients were injected with 2-deoxy-2-[^18^ F]fluoro-d-glucose ([^18^ F]-FDG) activities ranging between 205 and 263 MBq, followed by a 15 min data acquisition 30 min post-injection, hereby collecting between 118 and 145 M events.

### Image Reconstruction

All images were reconstructed using a list-mode time-of-flight ordered subsets expectation maximization (TOF-OSEM) algorithm following 3-class segmentation-based MRI-guided attenuation correction [19] with the exception of the Hoffman phantom data in which TOF information was not used. Scatter correction was performed using a TOF-dependent single scatter simulation technique. Images were reconstructed on a blob grid and subsequently sampled on a 288 × 288 × 90 image grid with a voxel size of 2 × 2 × 2 mm.

The image quality phantom scans were reconstructed using the combination of parameters displayed in Table 1. To cover all possible combinations, representative parameters were derived from the first (*u*_1_ = 6.988), second (*u*_2_ = 10.417), and third (*u*_3_ = 13.698) zero crossings. This resulted in 15 different parameter combinations, all satisfying Eq. 2. In all cases, the blob sampling distance was set at 2.0375. The data were reconstructed with various iterations (1, 2, 4, 8, and 12) using 32 subsets. For the Hoffman 3D brain phantom and the clinical studies, seven representative parameter combinations with 1, 4, and 12 iterations were used for reconstruction. These combinations are marked with a star in Table 1. The clinical studies were also reconstructed with the current protocol used in clinical routine, namely two iterations, an alpha value of 6.3716, a radius of 2.8, and blob spacing of 2.0375.

**Table 1:** Chosen radius parameters for given alpha values, used for the phantom studies satisfying conditions of Eq. 2. The blobs spacing (*d*) is set at 2.0375 (relative units)

Alpha	*r*_*1*_ Satisfying first zero crossing	*r*_*2*_ Satisfying second zero crossing	*r*_*3*_ Satisfying third zero crossing
1.3084	1.6302^*^	2.4074^*^	3.1552^*^
2.3664	1.6917	2.4495	3.1875
4.2799	1.8790	2.5824^*^	3.2907
7.7408	2.3912	2.9759	3.6078
10.4101	2.8750^*^	3.3769^*^	3.9451^*^

*combination used for Hoffmann brain phantom and clinical studies

### Evaluation Strategy

The image quality phantom was used to examine the influence of the blob parameters on the contrast and variance characteristics of the reconstructed images [20]. Image contrast is estimated by calculating the contrast recovery coefficient (CRC) for each sphere. Image variance is estimated through the image roughness (IR) and background variability (BV) metrics. IR reflects the variations within a region of interest (ROI) while BV reflects the variation between the background ROIs of the same size. Equations 3, 4, and 5 display the quantities involved in the calculation of these indices.

3 $$ CRC = \frac{\frac{m_{\mathrm{hr}}}{m_{\mathrm{r}}}-1}{t-1}*100 $$

where *m*_hs_ is the mean value of the hot sphere with size s at the target plane, *m*_s_ is the mean value of the background sphere with size s, *t* is the actual contrast recovery between the hot sphere and the background. In our experiment, *t* equals 5.

4 $$ I{R}_{\mathrm{s},\mathrm{k}} = \frac{\sqrt{\frac{1}{i-1}{\displaystyle \sum }{\left({f}_{\mathrm{i}}-{m}_{\mathrm{s},\mathrm{k}}\right)}^2}}{m_{\mathrm{s},\mathrm{k}}}*100 $$

where *m*_s,k_ is the mean value of the *k* ^*th*^ background ROI with size s, *i* is the pixel number, and *f*_i_ is the intensity of pixel i. To generate the final image roughness, the value is averaged over the 60 ROIs:

$$ I{R}_{\mathrm{s}}=\frac{1}{K}{\displaystyle \sum_{k=1}^K}I{R}_{\mathrm{s},\mathrm{k}}. $$

5 $$ B{V}_{\mathrm{s}} = \frac{\sqrt{\frac{1}{K-1}{\displaystyle \sum }{\left({m}_{\mathrm{s},\mathrm{k}}-\overline{m_s}\right)}^2}}{\overline{m_{\mathrm{s}}}}*100 $$

where *m*_s,k_ is the mean value of the *k* ^*th*^ background ROI with size *s* and $\overline{m_{\mathrm{s}}}=\frac{1}{K}{\displaystyle \sum_{k=1}^K}{m}_{\mathrm{s},\mathrm{k}}$.

To calculate these characteristics, a central slice containing all the hot spheres and four neighboring slices (10 and 20 mm from each side) were used. In each slice, 12 background ROIs were drawn, resulting in a total of 60 background ROIs for each sphere size. In the central slice, six hot sphere ROIs were also drawn. Supplemental Fig. 1a shows the ROIs overlaid on the target slice.

The Hoffman phantom was qualitatively and quantitatively analyzed. Quantitative analysis was performed using two indices: the radioactivity concentration ratio (RCR) and the contrast (C). The RCR (Eq. 6) evaluates the ratio between the estimated radioactivity concentration in the GM (*m*_GM_) and the WM (*m*_WM_).

6 $$ RCR=\frac{m_{\mathrm{GM}}}{m_{\mathrm{WM}}} $$

The contrast (Eq. 7) evaluates the contrast between the cold regions (cerebrospinal fluid (CSF) (*m*_csf_)) and the hot regions (*m*_GM_). The theoretical RCR for the Hoffman phantom is equal to 4 whereas the theoretical *C* is equal to 1.

7 $$ C=\frac{m_{\mathrm{GM}}-{m}_{\mathrm{csf}}}{m_{\mathrm{GM}}} $$

To calculate these characteristics, a central slice was selected at the level of the basal ganglia and the lateral ventricle, containing GM, WM, and CSF. Representative ROIs were then drawn on this slice (14 on the GM, nine on the WM, and four on the CSF) (Supplemental Fig. 1b).

The clinical studies were also evaluated qualitatively and quantitatively. Qualitative evaluation was performed blindly by two experienced nuclear medicine physicians (over 10 years experience). For each patient, the image reconstructed using optimal parameters was identified. The subsequent images reconstructed using different parameter combinations were visually graded based on overall image quality with a ranking system using a 4-step scale:

1. Excellent
2. Good
3. Moderate
4. Poor, not diagnostically useful

Quantitative evaluation was performed using the same indices used in the Hoffman phantom study, namely the RCR and C (Supplemental Fig. 1c).

## Results

### Image Quality Phantom

**Fig. 1:**
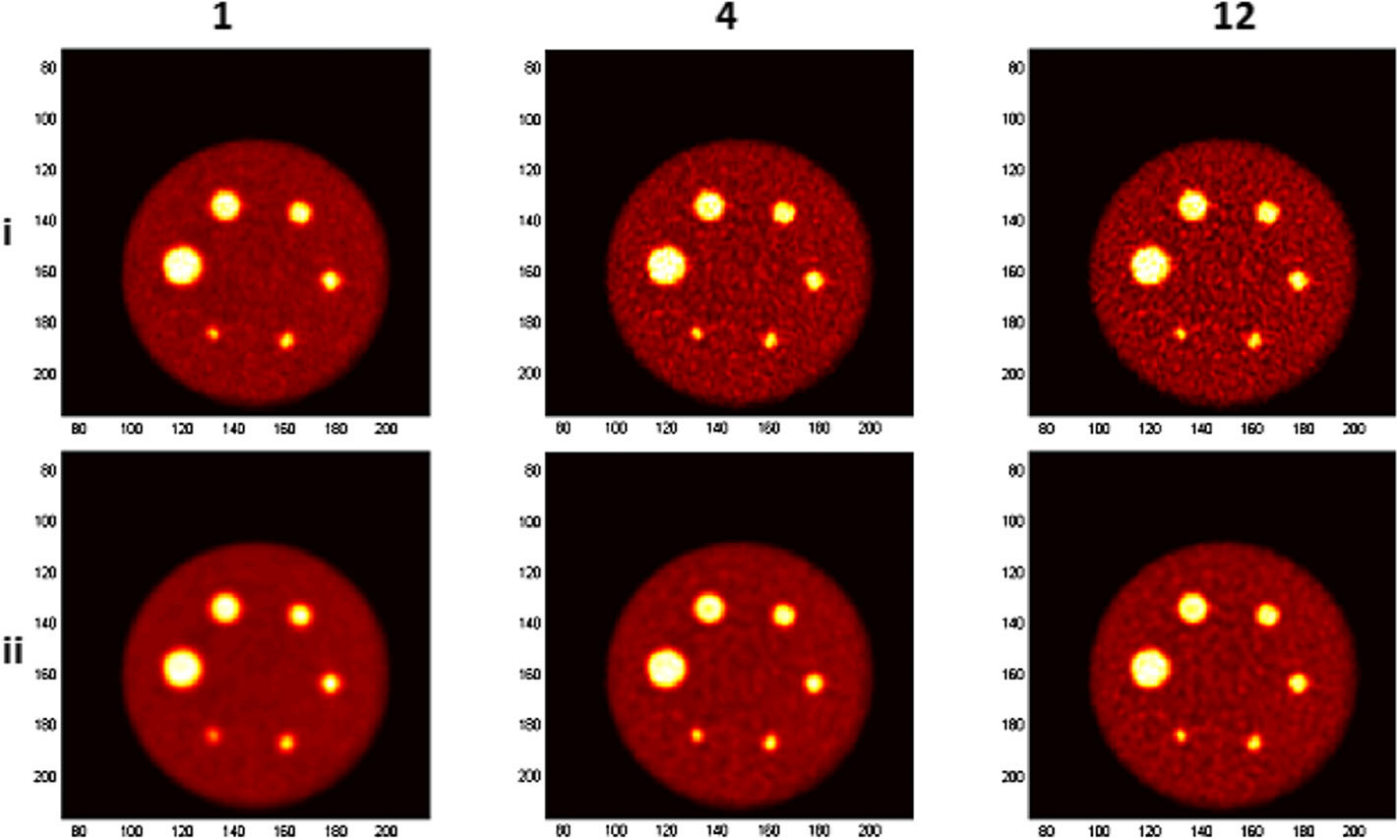
Reconstructed images centered at the hot spheres plane using from left to right, 1, 4, and 12 iterations with two parameter combinations: alpha = 1.3084, *r* = 1.6302, *d* = 2.0375 (*i*) and alpha = 10.4101, *r* = 3.9451, *d* = 2.0375 (*ii*).

**Fig. 2:**
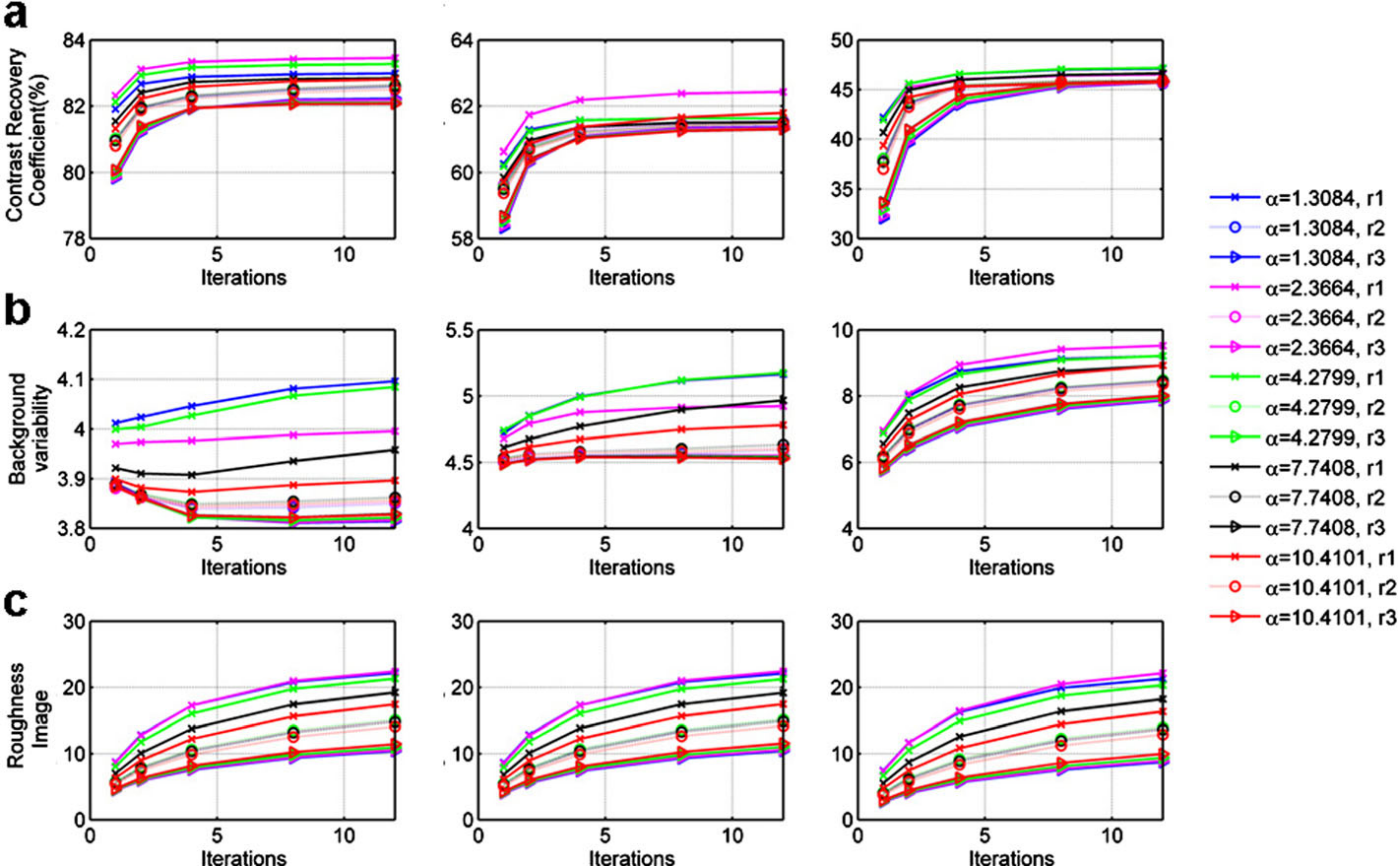
**a** Contrast recovery coefficients, **b** background variability, and **c** image roughness for a large (left), medium (middle), and small (right) sphere sizes using different reconstruction parameters. The different alpha values are represented by different colors while the first, second, and third zero crossing radius are defined by different *line styles*. The *y*-axis scale of each sphere size is adjusted for optimal representation.

Figure 1 shows reconstructed images using 1, 4, and 12 iterations for two extreme combinations of reconstruction parameters (alpha = 1.3084, *r* = 1.6302, *d* = 2.0375 and alpha = 10.4101, *r* = 3.9451, *d* = 2.0375). Visual inspection clearly shows different noise textures between iterations chosen parameter combinations. The amount of noise increases with iterations and the second combination of parameters (Fig. 1ii) showing lower noise levels compared to the first (Fig. 1i). Differences in contrast recovery are also observed between images reconstructed using different iterations and combination of parameters. The contrast slightly increases with increasing iterations, but overall the second set provides a lower contrast recovery. These differences, quantified in Fig. 2, are caused by the difference in the FWHM of the used blobs. The second set results correspond to a basis function with a larger FWHM, resulting in an increased smoothing of the images.

### Contrast Recovery Coefficient

Figure 2a shows the quantitative CRC values for three representative sphere sizes (smaller, middle, and larger sphere) based on different reconstruction parameters. As the sphere size increases, so does the CRC values. For the first parameter set and 12 iterations, the maximum CRC is 83 and 47 % for the large and small sphere, respectively. Absolute differences between parameter sets are small, approximately 2 % but several patterns could be recognized. The CRC decreases with larger radius values, but increases by 5–15 % with increasing iterations. Larger radius values result in a CRC decrease of approximately 1–2 % due to the fact that the blobs have a larger FWHM, also causing resolution degradation [15].

It can be observed that the reconstructions are grouped based on the radius representing first, second, and third zero crossings. In addition, the radii representing the first zero crossing have larger differences within the group. This finding is consistent with the errors found by Matej et al., with parameters in the first zero crossing resulting in errors up to 1 %, while the maximum error in the third crossing is substantially lower [11].

### Image Roughness and Background Variability

Figure 2b and c shows the calculated quantitative noise metrics; image roughness; and background variability (Eqs. 4 and 5). The BV values appear to decrease with sphere size. For smaller sphere sizes, larger BV variations are observed, substantiating that noise reduction is larger in smaller regions when using parameters from the third zero crossing. In contrast, IR is similar for all sphere sizes since the pixel to pixel variability depends less on sphere size.

For all sphere sizes, the IR increases by 5–15 % with iterations. The BV also show a small increase (0.3–3 %); however, for large sphere sizes and larger radii (third zero crossing), the BV stays almost constant. After 12 iterations, the largest difference between parameter sets is approximately 15 and 3 % for the IR and BV, respectively.

Similar groups are seen in both BV and IR as in the CRC results, representing the first, second, and third zero crossing. The variability within the first group is the largest (∼0.3–1 % and ∼5 % for BV and IR, respectively), the variability within the last group the smallest (0–0.25 % and 1 % for the BV and IR, respectively) probably due to larger errors for smaller blobs.

### Hoffmann Phantom Study

#### Visual Quality Assessment

**Fig. 3:**
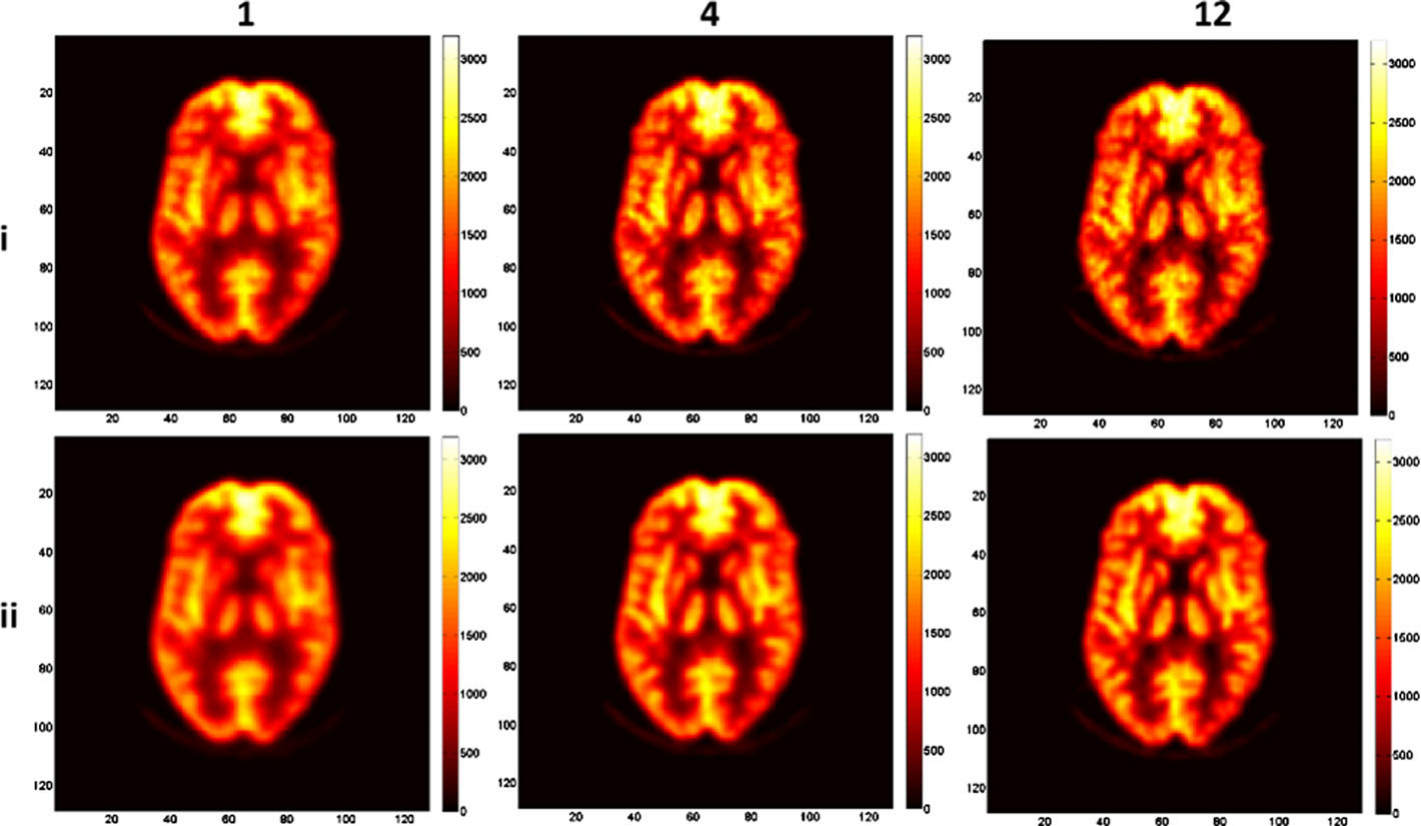
Reconstructed images of the Hoffman phantom with high count statistics using from left to right, 1, 4, and 12 iterations and two parameter combinations: alpha = 1.3084, *r* = 1.6302, *d* = 2.0375 (*i*) and alpha = 10.4101, *r* = 3.9451, *d* = 2.0375 (*ii*).

**Fig. 4:**
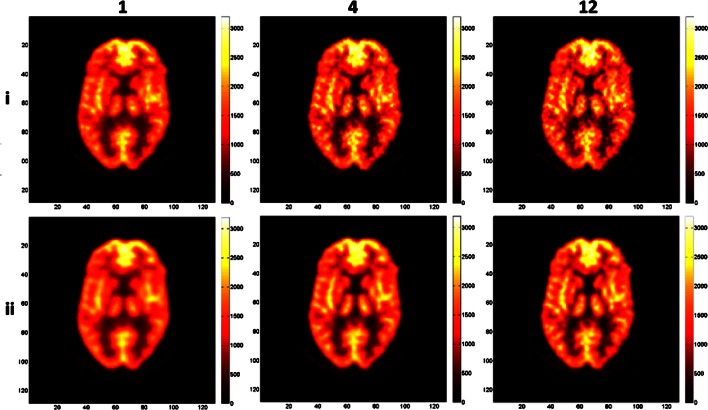
Reconstructed images of the Hoffman phantom with low count statistics using from left to right 1, 4, and 12 iterations with two parameter combinations: alpha = 1.3084, *r* = 1.6302, *d* = 2.0375 (*i*) and alpha = 10.4101, *r* = 3.9451, *d* = 2.0375 (*ii*).

Representative images of the Hoffman phantom with high and low count statistics are shown in Figs. 3 and 4, respectively. Similar differences are seen when using different iterations and parameter combinations as in the previous image quality phantom study. The images show an increased amount of noise with higher iterations and the second parameter set, with a larger radius and alpha values producing smoother and less noisy images owing to the larger FWHM of the blob. In all images, the anatomical structures could be recognized but their exact delineation is more straightforward in images reconstructed with a higher number of iterations. The images with low count statistics clearly show an increased noise texture. The recognition of anatomical boundaries is also more challenging.

#### Quantitative Analysis

**Fig. 5:**
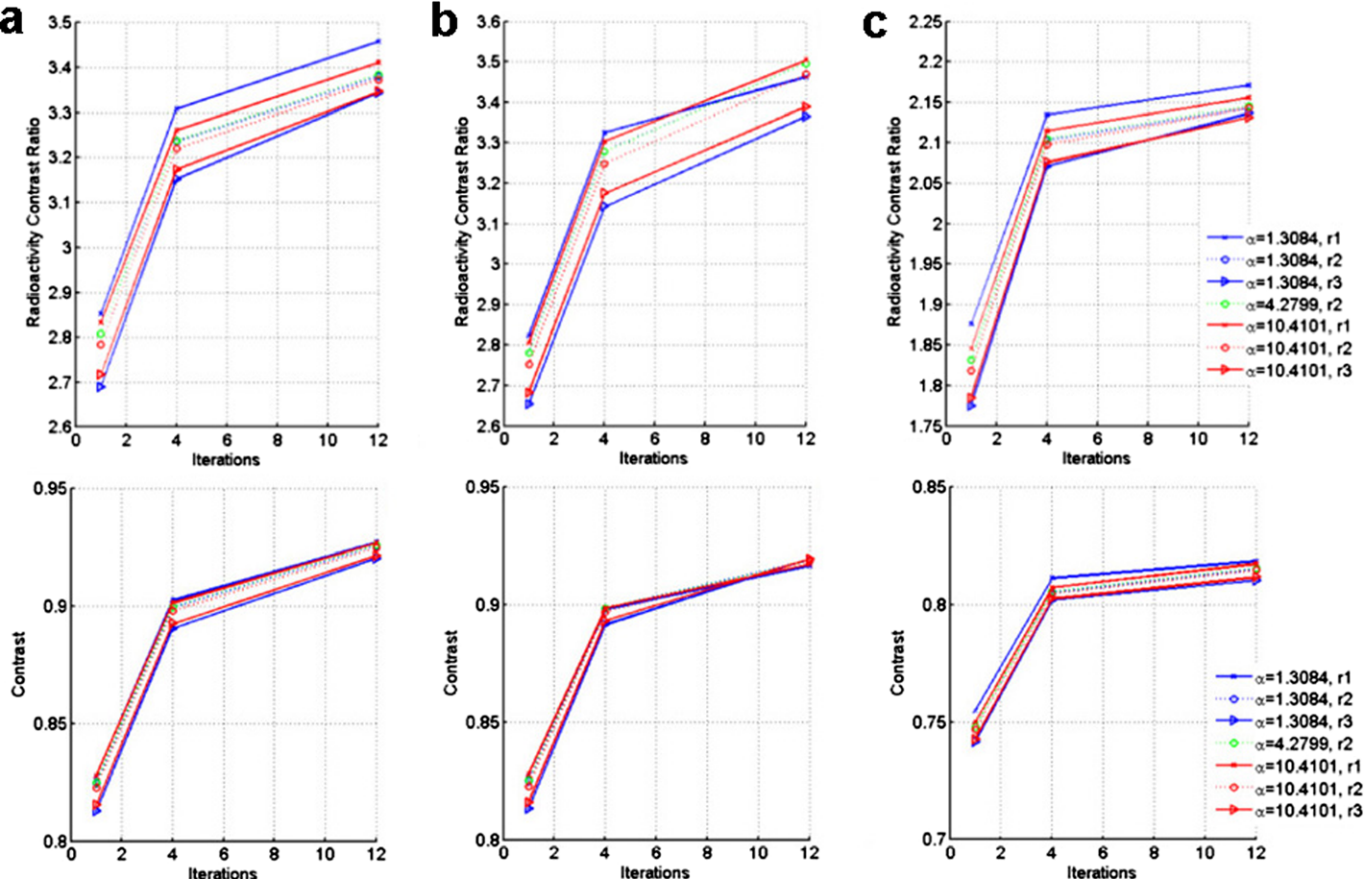
Radioactivity concentration ratio (*top*) and contrast for the Hoffman phantom (*bottom*) with **a** high and **b** low count statistics. Same metric for the clinical study shown in Fig. 6.

Two measures, representing the contrast between GM, WM, and the CSF, were used for quantitative analysis. The RCR and C are displayed in Fig. 5a and b, respectively. Theoretically, the RCR is equal to 4 whereas the C is equal to 1. High and low count statistics give comparable values for both metrics. It can be seen that both contrast ratios increase with higher iterations; the RCR increases from 2.7 to 3.5 and the C from 0.82 to 0.92. Again, grouping of the first and second zero crossing radii is observed. However, the differences between the groups are small (0.2 and 0.02 for the RCR and C, respectively).

### Clinical Studies

#### Comparison with Hoffman Phantom

**Fig. 6:**
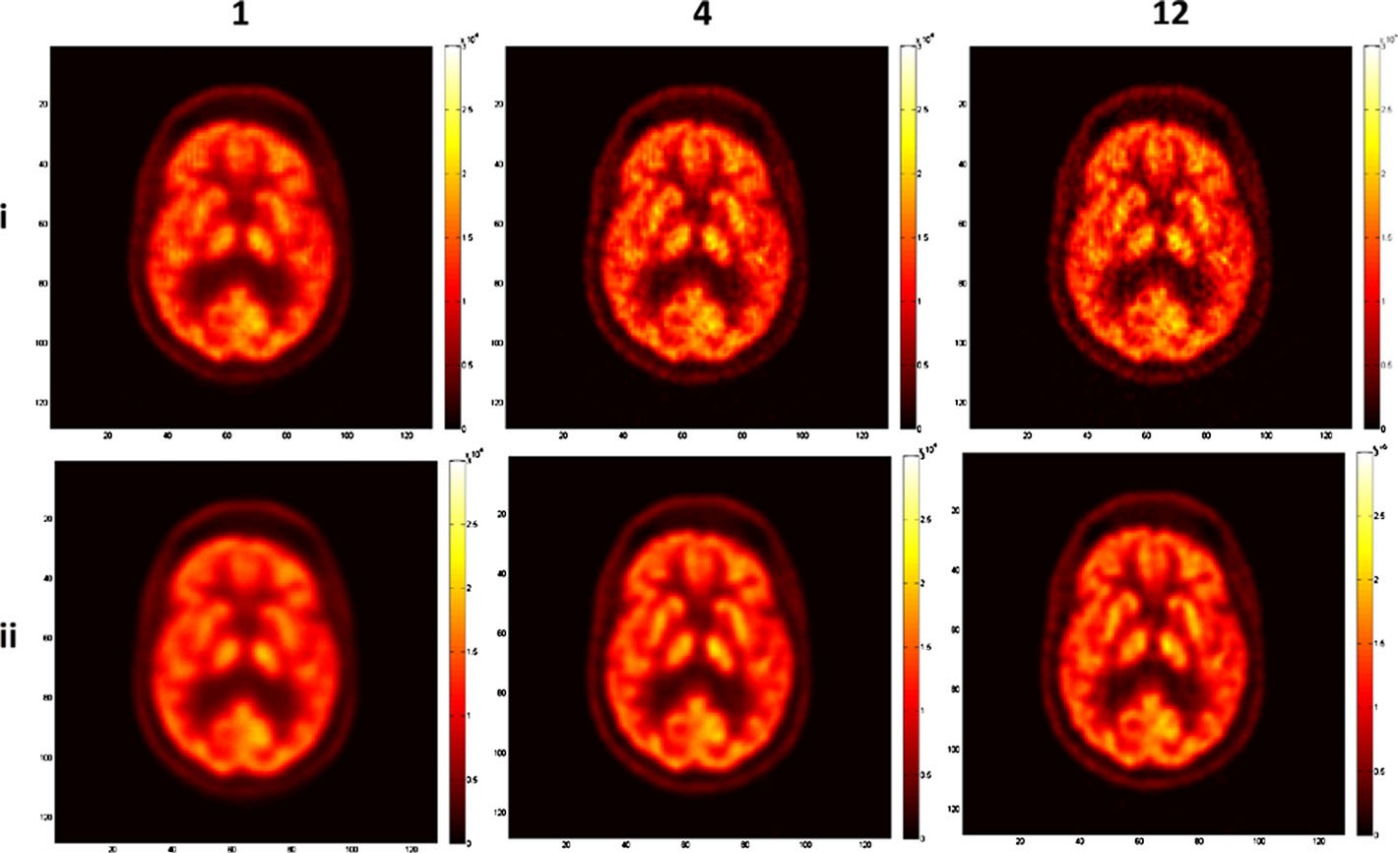
Reconstructed images of a clinical FDG-PET brain study using from left to right 1, 4, and 12 iterations with two parameter combinations: alpha = 1.3084, *r* = 1.6302, *d* = 2.0375 (*i*) and alpha = 10.4101, *r* = 3.9451, *d* = 2.0375 (*ii*).

Qualitative evaluation of a clinical FDG-PET brain study (Fig. 6) shows similar differences between reconstruction parameters and iterations as observed in the phantom studies. The overall activity distribution is similar to the Hoffman phantom but the basal ganglia show higher uptake. Moreover, the clinical images display similar noise texture as the low count Hoffman phantom images.

The quantitative evaluation of the clinical study is displayed in Fig. 5c. The RCR and C values are lower than the Hoffman phantom due to different and more complex activity distribution in the patients presenting with different neurological diseases. However, they follow the same trend, namely, the values increase with increasing iterations from 1.8 to 2.15 and from 0.74 to 0.82 for the RCR and C, respectively.

#### Visual Quality Assessment

Two physicians scored separately the clinical images blindly. Table 2 summarizes the frequencies of the ranking scores per observer. The overall observer agreement is 48 % but the second observer assigned lower scores more often. However, there was a moderate correlation between the two observers (spearman correlation coefficient of 0.661 (*p* < 0.0005)).

**Table 2:** Summary of the assessment of clinical studies by the two observers

	Observer 1					
Observer 2		Excellent	Good	Moderate	Poor	Total
	Excellent	0	0	0	0	0
	Good	8	15	2	0	25
	Moderate	5	20	19	1	45
	Poor	0	0	10	8	18
	Total	13	35	31	9	88

**Fig. 7:**
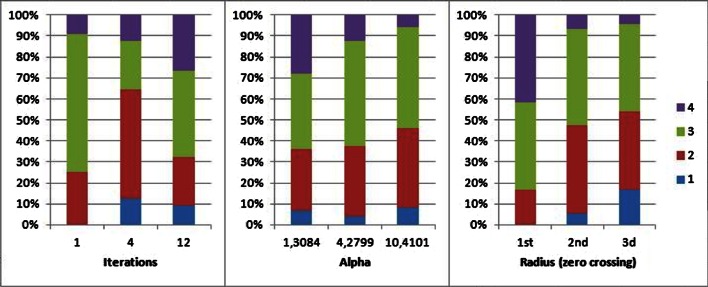
Distribution of scores as a function of iterations (left), alpha (middle), or radius (right). 1 is the highest score (excellent quality), 4 the lowest (poor quality).

Figure 7 summarizes the visual quality assessment as a function of the number of iterations, alpha, and radius. Several patterns could be recognized. The scores improve with increasing iterations as better scores were assigned. Higher scores were assigned to images reconstructed using four iterations than 12 iterations owing to lower variance characteristics of the reconstructed images. Larger radii and higher alpha values increased the scores. Thus, smooth images with lower noise texture and a slightly lower contrast were preferred.

A more detailed analysis of the results revealed that several scans scored better than the current clinical protocol (one time excellent, four times good, and three times moderate). The parameters corresponding to the scan with the best score were four iterations, alpha of 10.4101, and the third zero crossing (two times excellent and six times good).

## Discussion

This study aimed to achieve optimal image characteristics with an emphasis on brain PET imaging application and focusing on evaluating the contrast and noise properties for a variety of image reconstruction parameters. The Ingenuity TOF PET/MR system uses Kaiser–Bessel window function instead of conventional voxels to represent images during the reconstruction process. Blobs are controlled by a set of basis function parameters which influence the reconstructed image properties. Parameter optimization plays a pivotal role in the successful implementation and clinical use of blob-based reconstruction in PET imaging. Therefore, this study investigated the qualitative and quantitative image characteristics produced with a representative range of reconstruction parameters.

The phantom results show that larger blob radii better suppress the noise but produces lower contrast recovery. This pattern, observed in both phantom and clinical studies, is consistent with the root mean square error metric reported in previous studies [11, 21]. Although previous quantitative evaluation studies used only a few blob parameters, the overall results are consistent with those reported in this work, though the convergence rate was slower in the former studies [11, 12]. These discrepancies could be explained by differences in the iterative reconstruction algorithms used (the use of raw action maximum likelihood algorithm (RAMLA) instead of OSEM and different numbers of subsets) as well as their algorithmic implementations.

Absolute differences in contrast recovery are small, thus a larger blob with a slight decrease in CRC and increased noise suppression might be more optimal for visual evaluation. The visual interpretation of clinical studies by physicians showed a preference towards reconstructions using four iterations. Compared to higher number of iterations, these images have a slightly lower contrast but the image quality is improved due to lower noise levels. It is also observed that higher radii are preferred. Thus, in a clinical setting, lower variance levels are preferred over a slightly higher contrast. Overall, blob-based reconstructions using optimal parameters proved to produce images with a better visual quality compared to the current reconstruction protocol used in the clinic (data not shown).

These findings might depend on the type and pattern of tracer distribution in the organ of interest as well as on the type of information sought. In this work, we used FDG-PET brain studies with a high signal throughout the brain in order to identify diffuse patterns of hypometabolism, which are typical for neurodegenerative disorders. Other parameters might be more favorable in various indications using different tracers, such as aminoacidic tracers for characterization of focal brain lesions. One of the limitations of the current study is the relatively small number of clinical studies used for clinical evaluation by physicians. A larger cohort could potentially strengthen the findings of this work.

Spherical symmetric basis functions require longer computational time compared to traditional voxels due to overlapping basis functions. This study showed that all reconstructions up to four iterations are executed within 30 min and almost all combinations could be executed within an hour. These results are consistent with previous studies [13]. Therefore, all proposed parameter combinations could be used in a clinical setting. However, reconstruction times could be further optimized. Cabello et al. showed that the use of graphical processing unit (GPU) technology instead of central processing unit (CPU) technology was ∼four times faster [13]. Hu et al. followed a different approach to accelerate the reconstruction by ∼three times through exploitation of the scanner symmetry and alignment of the blob matrix with the crystal rings for LOR-based reconstruction [22].

This study only used the standard blob increment (d = 2.0375). A smaller increment could also be investigated at the expense of computational time. Previous studies reported resolution degradation for a basis function having a FWHM higher than the FWHM of the scanner [15]. This resolution degradation occurs sooner with a larger increment. Nevertheless, this work showed that for a range of blob parameters, the resulting images provide an acceptable visual and quantitative quality.

## Conclusion

A range of blob parameters hypothetically provide optimal image quality. The results of the phantom studies demonstrated that larger blob radii result in higher noise suppression but lower contrast recovery. Absolute contrast recovery differences are small, thus a larger blob with a slight decrease in CRC but with increased noise suppression might be more optimal. Although this work emphasized on brain imaging applications using the Ingenuity PET/MR, the findings could be applicable to other PET/MR and PET/CT systems using spherical symmetric elements for image representation. Furthermore, whole-body applications are also expected to benefit from this investigation as although certain parameters differ compared to brain applications (e.g., voxel size), these are not expected to substantially influence the underlying performance of the blob-based algorithms used. Finally, it should be emphasized that such an investigation should only be used as a guide and not as the *de facto* optimum solution. Ultimately, the optimal reconstruction parameters and the desired image characteristics should be evaluated on an application- and task-specific basis, depending on the quantitative or qualitative nature of the task.

## Electronic supplementary material

Below is the link to the electronic supplementary material.

ESM 1 (DOCX 449 kb)
