# Supplementary material for: Qualitative and Quantitative Evaluation of Blob-Based Time-of-Flight PET Image Reconstruction in Hybrid Brain PET/MR Imaging
Source: Mol Imaging Biol. 2015 Jan 30;17(5):704–13. doi: 10.1007/s11307-015-0824-x (PMC4768229; doi:10.1007/s11307-015-0824-x)
Supplement: Supplementary file 1 — (DOCX 449 kb) [file 11307_2015_824_MOESM1_ESM.docx]

**Qualitative and quantitative evaluation of blob-based time-of-flight PET image reconstruction in hybrid brain PET/MR imaging**

**Molecular Imaging and Biology**

Eva L. Leemans^1,2^, Fotis Kotasidis^1^, Michael Wissmeyer^1^, Valentina Garibotto^1^, Habib Zaidi^1,3,4†^

^1^Division of Nuclear Medicine and Molecular Imaging, Geneva University Hospital, CH-1211 Geneva 4, Switzerland

^2^Technical Medicine, University of Twente, Enschede, the Netherlands

^3^Geneva Neuroscience Center, Geneva University, CH-1205 Geneva, Switzerland

^4^Department of Nuclear Medicine and Molecular Imaging, University of Groningen, University Medical Center Groningen, 9700 RB Groningen, Netherlands

^†^**Corresponding author:**

Habib Zaidi, Ph.D

Geneva University Hospital

Division of Nuclear Medicine and Molecular Imaging

CH-1211 Geneva, Switzerland

**Tel:** +41 22 372 7258

**Fax:** +41 22 372 7169

**email:** habib.zaidi@hcuge.ch

**Short running title:** Evaluation of image reconstruction in brain PET

**Journal:** Molecular Imaging and Biology

**
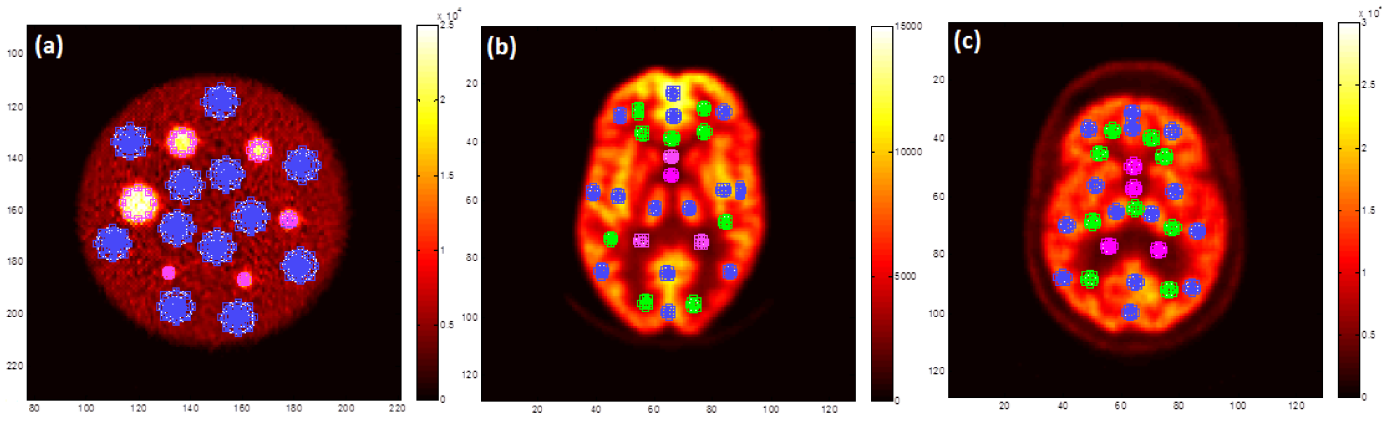
**

**Supplemental Figure 1.** ROI’s overlaid on the target slices **(**a) the image quality phantom, background (blue) and foreground (pink) ROI’s. (b) Hoffman 3D brain phantom, GM (blue), WM (green), CSF (pink) ROI’s. (c) Clinical FDG-PET brain scan, GM (blue), WM (green), CSF (pink) ROIs.
